# Herpes zoster and long-term risk of subjective cognitive decline

**DOI:** 10.1186/s13195-024-01511-x

**Published:** 2024-08-14

**Authors:** Tian-Shin Yeh, Gary C. Curhan, Barbara P. Yawn, Walter C. Willett, Sharon G. Curhan

**Affiliations:** 1 Department of Physical Medicine and Rehabilitation, School of Medicine, College of Medicine, Taipei Medical University, No.250, Wuxing St, Taipei, 11031 Taiwan; 2 Department of Physical Medicine and Rehabilitation, Wan Fang Hospital, Taipei Medical University, Taipei, Taiwan; 3 Department of Epidemiology and Nutrition, Harvard T. H. Chan School of Public Health, Harvard University, Boston, MA USA; 4 Channing Division of Network Medicine, Department of Medicine, Brigham and Women’s Hospital and Harvard Medical School, Boston, MA USA; 5 Department of Physical Medicine and Rehabilitation, National Taiwan University Hospital, Taipei, Taiwan; 6 Department of Physical Medicine and Rehabilitation, College of Medicine, National Taiwan University, Taipei, Taiwan; 7 Harvard Medical School, Boston, MA USA; 8 Renal Division, Department of Medicine, Brigham and Women’s Hospital, Boston, MA USA; 9 Department of Family and Community Health, University of Minnesota, Minneapolis, MN USA

**Keywords:** Herpes zoster, Shingles, Subjective cognitive decline, Vaccination, Immunocompromise, APOE ε4, Prospective cohort study

## Abstract

**Background:**

Herpes zoster (HZ), commonly known as “shingles,” may contribute to cognitive decline through mechanisms such as neuroinflammation or direct neuronal injury. However, evidence on the longitudinal association between HZ and cognitive decline is conflicting and whether the risk differs by APOE ε4-carrier status has not been studied; prospective cohort studies on the association between HZ vaccination and cognitive decline are also lacking.

**Methods:**

We included 149,327 participants from three large cohorts—the Nurses’ Health Study (NHS), NHSII, and Health Professionals Follow-Up Study (HPFS)—to prospectively examine the association between HZ and subsequent subjective cognitive decline (SCD). Poisson regression was used to estimate the multivariable-adjusted relative risk (MVRR) of a 3-unit increment in SCD score according to years since HZ compared with participants with no history of HZ.

**Results:**

Compared with individuals with no history of HZ, the MVRR (95% CI) of a 3-unit increment in SCD score was significantly and independently higher among individuals with a history of HZ, but the duration of time since HZ when the elevated risk of SCD was statistically significant differed among the cohorts. In NHS, HZ was associated with higher long-term risk of SCD; compared with individuals with no history of HZ, the MVRR (95% CI) of a 3-unit increment in SCD score was 1.14 (1.01, 1.32) for ≥ 13 years since HZ. In NHS II, HZ was associated with higher risk of SCD in both the short-term [MVRR 1.34 (1.18, 1.53) for 1–4 years] and long-term [MVRR 1.20 (1.08, 1.34) for ≥ 13 years since HZ]. In HPFS, an elevated risk of SCD was suggested across all time points. Among the subset of participants with information on APOE ε4, there was a suggestion that the association differed by APOE ε4 carrier status, but the results were not consistent between women and men. Among the subset of women with information on HZ vaccination, there was a suggestion that the long-term risk of SCD may be greater among women who were not vaccinated against HZ.

**Conclusions:**

Data from three large independent cohorts of women and men showed that HZ was associated with higher long-term risk of SCD, and the risk may differ by APOE ε4-carrier status.

**Supplementary Information:**

The online version contains supplementary material available at 10.1186/s13195-024-01511-x.

## Introduction

In the era of rapid population aging, age-related cognitive decline poses a significant global burden and warrants further investigation to better understand its risk factors and potential interventions [1, 2]. There is growing evidence that herpesviruses may influence the risk of cognitive decline and dementia [3]. Herpes zoster (HZ), commonly known as “shingles,” is caused by reactivation of the neurotrophic varicella-zoster virus (VZV). VZV is a double-stranded DNA virus that causes varicella (chickenpox) and then establishes lifelong latency in ganglionic neurons in > 95% of Americans [4]. VZV reactivation from ganglia and spread to one or more corresponding dermatomes results in HZ, and affects one in three individuals during their lifetime [5]. HZ may potentially contribute to dementia risk through neuroinflammation, cerebral vasculopathy, or direct neuronal damage [6], but current evidence regarding the relationship between HZ and subsequent risk of cognitive decline is conflicting. Some studies found that HZ was associated with a higher risk of Alzheimer’s disease (AD) [7–9], but some found no association [10, 11], whereas others indicated that HZ was associated with an unexpected lower risk of dementia [12, 13], and suggested that VZV vaccination may not reduce dementia risk [13]. Although protective associations between HZ vaccination and dementia have been reported [14], existing literature primarily consists of retrospective studies or case-control studies [10, 15], and high-quality prospective cohort studies are needed to deepen the understanding of this topic. Carriage of another herpes virus, herpes simplex virus (HSV1/2), was associated with greater decline in episodic memory function, and a significant interaction between APOE ɛ4-carrier status and HSV1/2 for episodic memory decline was demonstrated (p for interaction < 0.001) [16]. In addition, among individuals who were APOE ε4 carriers, reactivation of herpes simplex virus type 1 (HSV-1) was associated with higher risk of Alzheimer’s disease (AD), but no association was found between HSV-1 and AD among those who were APOE ε4 non-carriers [17]. However, to our knowledge, whether the association of HZ and SCD differs by APOE ε4-carrier status remains unexplored.

Subjective cognitive decline (SCD) is a measure of early changes in cognition that captures an individual’s self-perceived experience of cognitive decline even before evidence of cognitive impairment may be apparent on standardized neuropsychological tests [18, 19]. Among individuals with SCD, approximately 7% will progress to dementia and 21% to mild cognitive impairment (MCI) [20]. The risk of dementia is 2.17 times for patients with SCD compared to those without [21]. Given the high risk of progression from SCD to MCI and dementia, understanding the association between HZ and subsequent SCD may be crucial for the early detection and prevention of cognitive decline in individuals at risk, and may provide important insight into the relation of VZV and dementia.

Therefore, we prospectively examined the long-term association between HZ and the risk of SCD using data from three large well-characterized cohorts of women and men—the Nurses’ Health Study (NHS), NHSII, and Health Professionals Follow-Up Study. We also examined whether the association of HZ and SCD varied by *APOE* e4 carrier status, HZ vaccination status, or immunocompromised status.

## Methods

### Study population

The Nurses’ Health Study (NHS) started in 1976 when 121,700 female registered nurses aged 30–55 years were recruited and completed a baseline questionnaire. The NHSII began in 1989 with 116,429 female registered nurses aged 25–42 years. The Health Professionals Follow-Up Study (HPFS) began in 1986 when 51,529 male US health professionals aged 40–75 years were enrolled. Follow-up questionnaires have been sent to the participants every two years to obtain detailed information on medical history, newly diagnosed diseases, and potential risk factors; the > 30-year follow-up exceeds 90% of eligible person-time. For the current study, the baseline was defined as the year when information on the time of HZ occurrence was first available. Participants whose HZ date was unavailable were excluded from the analyses.

### Herpes zoster (HZ) ascertainment

Information on HZ, including the date of occurrence, was collected in the years 2000, 2004, 2008, and 2012 for the NHS; in 2001, 2005, 2013, and 2017 for the NHSII; and in 2004, 2006, and 2008 for the HPFS. To ascertain the validity of the self-reported HZ information, we conducted a validation study comparing reports of HZ by questionnaire with medical records. We requested permission to obtain medical records related to the HZ event from a randomly selected subset of NHS and NHSII participants who reported a history of HZ on the 2021 biennial questionnaire. In the subset of participants for whom medical records were obtained, a diagnosis of herpes zoster was confirmed in 230/231 (99.6%), indicating high reliability of self-reported HZ. For the primary analysis, we categorized the exposure according to years since the participant’s HZ event; participants without HZ history served as the reference group.

### Assessment of subjective cognitive decline (SCD)

SCD was assessed in 2012 and 2014 for the NHS, 2008 and 2012 for the HPFS, and 2017 for NHSII. For the HPFS, the SCD scores were based on the following 6 yes/no questions: [1] “Do you have more trouble than usual remembering a short list of items, such as a shopping list?”; [2] “Do you have more trouble than usual remembering recent events?”; [3] “Do you have trouble remembering things from one second to the next?”; [4] “Do you have any difficulty in understanding things or following spoken instructions?”; [5] “Do you have trouble finding your way around familiar streets?” and [6] “Do you have more trouble than usual following a group conversation or a plot in a TV program due to your memory?” For the NHS and NHSII, the SCD scores had one additional question: “Have you recently experienced any change in your ability to remember things?” [22] One point was given for each yes, all questions had equal value. For participants who had two SCD assessments, we averaged the two scores to reduce random error; for participants who only had one SCD assessment, the single score was used for analysis.

A previous study demonstrated strong associations between SCD and both concurrent objective cognitive function [22] and subsequent cognitive decline [22], which supports the validity of SCD. SCD was also strongly associated with *APOE* ε4 genotype in both the NHS and HPFS, which further strengthens its validity [23]. In addition, SCD may be a stronger predictor of longitudinal cognitive decline in those with higher educational attainment [24]. A number of risk factors for dementia, including hypertension, hyperlipidemia, type 2 diabetes, cardiovascular disease, and heavy smoking, were shown to be related to higher risk of SCD [22].

### Covariates

In all three cohorts, data on covariates of interest were collected prospectively on the baseline and follow-up questionnaires. In our multivariable-adjusted models, we adjusted for the following covariates: age, race (white, black, other), family history of dementia, smoking (pack-years), alcohol consumption (g per day), body mass index (BMI) (kilograms/meters^2^), physical activity (metabolic equivalents, MET-hours/week), diabetes, hypertension, elevated cholesterol, stroke, coronary heart disease (CHD) (non-fatal/fatal myocardial infarction, fatal CHD, or coronary revascularization procedure), dietary quality (based on the Alternative Healthy Eating Index [AHEI]-2010 Score), depression (defined as anti-depressant use or self-reported depression), and self-reported medical conditions that potentially compromise immunity because of disease or treatment (e.g., cancer other than nonmelanoma skin cancer, rheumatoid arthritis (RA), Crohn’s disease (CD), ulcerative colitis (UC), systemic lupus erythematosus (SLE), asthma, diabetes, chronic obstructive pulmonary disease (COPD), and oral steroid use). For women, information on menopausal status and menopausal hormone therapy use, census tract income (<$50,000, $50,000–74,999, or >=$75,000/y), and husband’s education (high school or lower education, college, graduate school) were available. HZ vaccination status was available for NHSII. For men, we further adjusted for profession (dentist, pharmacist, optometrist, osteopath, podiatrist, veterinarian).

### Statistical analysis

Time since HZ was categorized as never, 1–4 years since HZ, 5–8 years since HZ, 9–12 years since HZ, and ≥ 13 years since HZ. We calculated the age-standardized characteristics of participants according to years since the HZ event. Consistent with methods previously used in these cohorts, Poisson regression was used to investigate the association between HZ and SCD because of the nature and distribution of the SCD scores [25]. The SCD score is a discrete count that follows a distribution with a non-negative integer range (0–6 for HPFS and 0–7 for NHS and NHSII) [22]. Due to the right-skewed SCD scores, Poisson regression is a useful statistical method specifically designed for analyzing count data with this property that provides a straightforward interpretation of the relative risk per unit increase in y (SCD). Because three or more positive SCD questions suggest poor cognitive function [22, 26], relative risks (RRs) and 95% confidence intervals (CIs) for a 3-unit increment in SCD were calculated [27]. Due to the non-linear relation between age and SCD, both a linear term and a quadratic term for age were included in all models [28]. For data on covariates for which we did not have information due to the information was missing or reported as ‘unknown’, a missing/unknown category was included in the multivariate model using an indicator variable for each covariate. The percentage of missing/unknown values for the individual covariates was very low (0 to < 5%), except for husband’s education in the female cohorts and family history of dementia in NHS.

We conducted stratified analyses among those with and without health conditions or treatments that could potentially compromise immunity. In addition, we conducted analyses stratified according to APOE ε4 allele carrier status (yes/no) among participants whose APOE ε4 was measured or imputed in previous genome-wide association studies [29]. For the NHSII, we further adjusted for HZ vaccination status and we also performed analyses stratified by HZ vaccination status. SAS software, version 9.4 (SAS Institute Inc., Cary, NC) was used for all analyses.

## Results

There were a total of 149,327 participants, including 56,142 women in the NHS (mean [SD] age 64.6 [6.7] years at baseline), 66,966 women (47.2 [5.3] years) in the NHSII, and 26,219 men (68.5 [8.0] years) in the HPFS. The age-standardized characteristics of study participants according to years since HZ are shown in Table 1. Men with a history of HZ were slightly older than those with no history of HZ. In all three cohorts, participants with a history of HZ were more likely to have depression or other conditions that could potentially compromise immunity, including cancer, RA, CD, UC, SLE, asthma, COPD, and oral steroid use. The frequencies of participants with SCD ≥ 3 according to time since HZ in the three cohorts are shown in Supplementary Fig. 1.

**Fig. 1:**
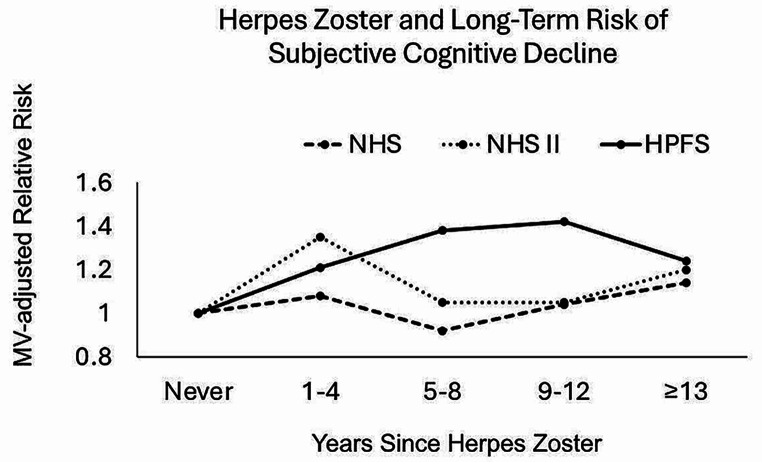
Herpes Zoster and Multivariable-Adjusted Relative Risk of 3 Unit Increment in Subjective Cognitive Decline in the Nurses’ Health Study (NHS), the Nurses’ Health Study II (NHS II), and the Health Professionals Follow-Up Study (HPFS). **NHS, NHS II**: Multivariable models adjusted for adjusted for age, race, family history of dementia, census tract income, husband’s education, smoking history, alcohol consumption, body mass index, physical activity, diabetes mellitus, hypertension, elevated cholesterol, diet quality (AHEI-2010 Score), menopausal status, depression, post-menopausal hormonal therapy use, herpes zoster vaccination (NHS II, only), and potentially immunocompromising conditions or treatments [a report of one or more of the following: cancer (other than non-melanoma skin cancer), rheumatoid arthritis, Crohn’s disease/ulcerative colitis (inflammatory bowel disease), systemic lupus erythematosus, asthma, chronic obstructive pulmonary disease, oral steroids/corticosteroid use), stroke, and coronary heart disease]. **HPFS**: Multivariable model adjusted for age, race, family history of dementia, smoking history, alcohol consumption, body mass index, physical activity, diabetes mellitus, hypertension, elevated cholesterol, diet quality (AHEI-2010 Score), depression, profession, and potentially immunocompromising conditions or treatments [a report of one or more of the following: cancer (other than non-melanoma skin cancer), rheumatoid arthritis, Crohn’s disease/ulcerative colitis (inflammatory bowel disease), systemic lupus erythematosus, asthma, chronic obstructive pulmonary disease, oral steroids/corticosteroid use), stroke, and coronary heart disease

A history of HZ was significantly and independently associated with higher long-term risk of SCD in both women and men (Table 2; Fig. 1). The duration of time since the episode of HZ when a significant elevation in risk of SCD was observed differed between the 3 cohorts. In NHS, HZ was associated with higher long-term risk of SCD; compared with individuals with no history of HZ, the MVRR (95% CI) of a 3-unit increment in SCD score was 1.14 (1.01, 1.32) for ≥ 13 years since HZ. In NHS II, HZ was associated with higher risk of SCD in both the short-term [MVRR 1.34 (1.18, 1.53) for 1–4 years] and long-term [MVRR 1.20 (1.08, 1.34) for ≥ 13 years since HZ]. In HPFS, an elevated risk of SCD was suggested across all time points. In multivariable-adjusted models that did and did not include stroke and CHD, the results did not materially differ (Supplementary Table 1). For all three cohorts, the point estimates were most attenuated by the adjustment for depression, compared with all other covariates (Supplementary Table 1).

**Table 1:** Age-standardized baseline characteristics of women in the Nurses’ Health Study I (NHS I), NHS II, and men in the Health Professionals Follow-Up Study (HPFS)

	Years since herpes zoster (HZ)				
**NHS I**	Never (*n* = 48,158)	1–4 years (*n* = 1701)	5–8 years (*n* = 2509)	9–12 years (*n* = 2193)	13 + years (*n* = 1581)
Age, years	64.5 (6.7)	64.6 (6.6)	65.0 (6.6)	65.4 (6.7)	65.6 (6.9)
Race, white, %	97.3	97.6	98.0	98.4	98.2
Body mass index, kg/m²	26.9 (5.3)	27.0 (5.4)	26.6 (5.0)	27.1 (5.4)	26.9 (5.2)
Physical activity, METs/week	18.7 (22.5)	17.9 (21.8)	18.3 (21.3)	17.7 (20.2)	18.3 (21.4)
Pack-years of smoking					
-Never smoker, %	46.5	48.0	44.2	45.7	44.7
-1-24 pack-years, %	10.7	10.4	12.3	10.0	11.4
-25-44 pack-years, %	22.8	21.1	22.6	23.7	24.3
-45 + pack-years, %	18.4	18.5	19.4	19.4	18.1
AHEI-2010 score	52.1 (9.3)	51.7 (9.4)	52.0 (9.1)	52.4 (9.0)	52.0 (9.3)
Alcohol intake, g/day	5.5 (8.3)	5.3 (7.8)	5.9 (8.6)	5.2 (7.7)	5.5 (8.2)
History of cancer, %	13.5	14.8	15.4	14.2	16.0
Rheumatoid arthritis, %	8.2	9.7	9.5	11.0	9.7
Crohn’s disease or Ulcerative colitis, %	1.9	1.6	2.7	2.2	2.2
Systemic lupus erythematosus, %	0.9	1.0	1.3	1.2	1.5
Asthma, %	10.5	11.4	11.1	12.3	10.8
COPD, %	3.8	4.4	4.1	4.4	5.1
Oral steroids use, %	1.9	2.2	2.5	2.6	3.8
Hypertension, %	46.2	48.2	46.9	47.4	45.4
Diabetes, %	7.5	7.4	6.5	7.4	7.7
Elevated cholesterol, %	58.6	60.1	61.3	61.3	61.8
Post-menopausal, %	98.5	98.8	98.4	99.1	98.7
Post-menopausal hormonal therapy use, ever, %	72.2	74.3	74.9	75.1	73.3
Depression, %	14.0	15.9	14.4	16.3	15.2
Family history of dementia, %	19.8	20.1	21.0	20.6	20.4
Husband’s education					
-<=high school, %	32.1	33.3	31.8	33.5	32.0
-college graduate, %	22.6	21.5	25.0	24.6	24.3
-graduate school, %	19.7	20.2	20.8	20.5	21.3
Census track income					
-<$50K, %	27.2	28.3	26.8	27.4	25.5
-$50K to <$75K, %	45.1	43.7	44.7	46.8	46.7
-$75K+, %	27.6	27.9	28.5	25.8	27.4
**NHS II**	Never (*n* = 57,585)	1–4 years (*n* = 2056)	5–8 years (*n* = 2595)	9–12 years (*n* = 1363)	13 + years (*n* = 3367)
Age, years	47.1 (5.4)	47.3 (5.3)	47.7 (4.9)	47.9 (4.7)	47.8 (5.0)
Race, white, %	94.7	95.0	95.1	95.9	95.6
Body mass index, kg/m²	26.7 (6.2)	26.9 (6.3)	26.9 (6.4)	27.3 (6.3)	27.3 (6.5)
Physical activity, METs/week	21.5 (27.2)	20.7 (26.9)	20.0 (25.5)	21.2 (34.2)	19.8 (26.2)
Pack-years of smoking					
-Never smoker, %	66.1	65.9	64.6	64.4	64.3
-1-24 pack-years, %	28.2	27.8	29.6	29.0	28.8
-25-44 pack-years, %	4.5	5.2	4.6	5.2	5.5
-45 + pack-years, %	0.5	0.4	0.6	0.8	0.8
AHEI-2010 score	50.0 (9.9)	50.0 (10.0)	50.1 (10.0)	50.2 (9.8)	49.8 (9.8)
Alcohol intake, g/day	3.7 (6.0)	3.6 (6.0)	3.7 (6.2)	3.5 (6.0)	3.5 (5.6)
History of cancer, %	5.1	5.1	5.3	5.1	5.9
Rheumatoid arthritis, %	2.3	3.5	3.0	2.6	3.9
Crohn’s disease or Ulcerative colitis, %	1.2	1.3	1.5	1.5	2.4
Systemic lupus erythematosus, %	0.4	1.0	0.5	1.0	1.1
Asthma, %	12.4	13.7	14.9	14.2	16.9
COPD, %	1.2	1.7	1.2	1.4	2.0
Oral steroids use, %	1.3	2.4	1.7	2.1	2.9
Hypertension, %	18.2	20.4	20.3	19.6	21.2
Diabetes, %	6.6	7.6	6.5	6.6	7.4
Elevated cholesterol, %	31.9	33.8	33.9	33.8	37.0
Post-menopausal, %	26.4	27.9	27.5	28.5	31.0
Post-menopausal hormonal therapy use, ever, %	0.2	0.2	0.1	0.2	0.2
Depression, %	20.0	21.9	21.2	24.2	24.8
Family history of dementia, %					
Husband’s education					
-<=high school, %	15.1	15.8	16.2	16.4	16.5
-college graduate, %	43.5	43.9	42.2	42.6	42.4
-graduate school, %	27.0	26.0	28.0	26.3	25.1
Census track income					
-<$50k, %	11.7	11.7	12.9	12.1	13.7
-$50k to <$75k, %	20.7	20.9	21.1	22.9	23.0
-$75k+, %	43.6	44.3	45.7	44.7	42.9
**HPFS**	Never (*n* = 23,722)	1–4 years (*n* = 830)	5–8 years (*n* = 518)	9–12 years (*n* = 437)	13 + years (*n* = 712)
Age, years	68.3 (8.0)	70.2 (8.0)	70.3 (7.8)	70.5 (8.0)	70.8 (8.2)
Race, white, %	91.6	93.3	92.5	91.9	92.2
Body mass index, kg/m²	26.2 (3.7)	26.0 (3.3)	26.1 (3.5)	25.6 (3.3)	25.9 (3.7)
Physical activity, METs/week	41.2 (35.0)	40.9 (33.9)	43.5 (37.2)	40.3 (34.9)	43.8 (35.4)
Pack-years of smoking					
- never smoker, %	49.7	48.1	47.9	50.2	46.3
- 1–4 pack-years, %	28.7	30.6	28.9	27.7	30.9
- 5–24 pack-years, %	11.2	10.8	13.7	10.4	10.4
- 25 + pack-years, %	5.1	5.5	4.1	5.8	6.1
AHEI-2010 score	55.1 (10.0)	54.9 (10.0)	55.2 (10.2)	56.3 (9.9)	55.4 (10.3)
Alcohol intake, g/day	11.2 (12.9)	10.6 (12.4)	11.5 (12.8)	10.7 (12.2)	11.1 (12.7)
History of cancer, %	19.2	20.2	19.3	19.0	20.5
Rheumatoid arthritis, %	6.7	8.9	7.0	6.9	7.6
Crohn’s disease or Ulcerative colitis, %	2.1	2.0	2.7	3.7	1.8
Asthma, %	9.2	11.4	9.5	10.6	8.3
COPD, %	3.0	4.2	5.0	3.1	3.6
Oral steroids use, %	1.2	2.2	1.6	2.2	1.9
Hypertension, %	49.6	52.9	52.3	52.1	49.0
Diabetes, %	9.1	10.5	10.5	10.4	9.7
Elevated cholesterol, %	60.2	62.3	61.8	60.7	65.6
Depression, %	19.9	21.4	23.8	23.3	26.5
Family history of dementia, %	21.0	22.4	17.9	27.4	19.7
Profession					
- Dentist, %	57.9	54.2	56.7	54.3	53.9
- Pharmacist, %	8.3	10.1	8.7	6.4	9.2
- Optometrist, %	6.7	7.6	6.4	8.7	7.6
- Osteopath, %	3.9	4.4	5.4	5.9	2.9
- Podiatrist, %	2.5	2.2	2.5	2.5	3.3
- Veterinarian, %	20.7	21.6	20.5	22.2	23.1

Values are means (SD) for continuous variables; Except for age at baseline, values of means or percentages are standardized to the age distribution of the study population

Not all categories add up to 100% due to rounding or information unknown

COPD: chronic obstructive pulmonary disease

**Table 2:** Herpes zoster and RR(95% CI) of 3 unit increment in Subjective Cognitive Decline (SCD) in the Nurses’ Health Study (NHS), the Nurses’ Health Study II (NHS II), and the Health Professionals Follow-Up Study (HPFS)

	Years Since Herpes Zoster				
	Never	1–4 years	5–8 years	9–12 years	≥ 13 years
**NHS**					
	(*n* = 48,158)	(*n* = 1701)	(*n* = 2509)	(*n* = 2193)	(*n* = 1581)
Age-adjusted RR (95% CI)	1.00 (ref)	1.15 (0.99, 1.32)	0.96 (0.86, 1.08)	1.09 (0.97, 1.23)	1.22 (1.06, 1.40)
MV RR^a^ (95% CI)	1.00 (ref)	1.08 (0.94, 1.24)	0.92 (0.82, 1.03)	1.04 (0.92, 1.17)	1.14 (1.01, 1.32)
**NHS II**					
	(*n* = 57,585)	(*n* = 2056)	(*n* = 2595)	(*n* = 1363)	(*n* = 3367)
Age-adjusted RR(95% CI)	1.00 (ref)	1.42 (1.25, 1.63)	1.10 (0.97, 1.25)	1.12 (0.95, 1.33)	1.38 (1.24, 1.53)
MV RR^a^ (95% CI)	1.00 (ref)	1.35 (1.18, 1.54)	1.05 (0.93, 1.19)	1.05 (0.89, 1.24)	1.20 (1.08, 1.33)
MV RR^b^ (95% CI)	1.00 (ref)	1.34 (1.18, 1.53)	1.05 (0.93, 1.19)	1.05 (0.89, 1.24)	1.20 (1.08, 1.34)
**HPFS**					
	(*n* = 23,722)	(*n* = 830)	(*n* = 518)	(*n* = 437)	(*n* = 712)
Age-adjusted RR (95% CI)	1.00 (ref)	1.29 (1.03, 1.62)	1.47 (1.11, 1.95)	1.61 (1.19, 2.18)	1.42 (1.11, 1.82)
MV RR^a^ (95% CI)	1.00 (ref)	1.21 (0.97, 1.53)	1.38 (1.04, 1.84)	1.42 (1.04, 1.92)	1.24 (0.97, 1.58)

^a^Multivariable model: NHSI & NHSII adjusted for age, race, family history of dementia, census tract income, husband’s education, smoking history, alcohol consumption, body mass index (BMI), physical activity, diabetes mellitus, hypertension, elevated cholesterol, Alternate Healthy Eating Index (AHEI-2010) Score, menopausal status, depression, post-menopausal hormonal therapy use, potentially immunocompromising conditions or treatments (a report of one or more of the following: cancer (other than non-melanoma skin cancer), rheumatoid arthritis (RA), Crohn’s disease/ulcerative colitis (inflammatory bowel disease), systemic lupus erythematosus (SLE), asthma, chronic obstructive pulmonary disease (COPD), oral steroids/corticosteroid use), stroke, and coronary heart disease (CHD);

HPFS adjusted for age, race, family history of dementia, smoking history, alcohol consumption, body mass index (BMI), physical activity, diabetes mellitus, hypertension, elevated cholesterol, Alternate Healthy Eating Index (AHEI-2010) Score, depression, profession, potentially immunocompromising conditions or treatments (a report of one or more of the following: cancer (other than non-melanoma skin cancer), rheumatoid arthritis (RA), Crohn’s disease/ulcerative colitis (inflammatory bowel disease), systemic lupus erythematosus (SLE), asthma, chronic obstructive pulmonary disease (COPD), oral steroids/corticosteroid use), stroke, and coronary heart disease (CHD)

^b^Multivariable model further adjusted for herpes zoster vaccination status

NHS: Nurses’ Health Study

NHS II: Nurses’ Health Study II

HPFS: Health Professionals Follow-Up Study

MV RR: Multivariable-adjusted relative risk

CI: Confidence Interval

In stratified analyses, we did not observe consistent differences in the association between HZ and long-term risk of SCD among those with and without immunocompromising conditions (p-for-interaction = 0.8 in NHS, 0.06 in NHS II, and > 0.99 in HPFS) (Table 3). In analyses stratified by *APOE* ε4 allele carrier status, there was a suggestion that the risk of SCD was significantly elevated among women who were non-carriers of the *APOE* ε4 allele, but the p-for-interaction was not significant in either female cohort (p-interaction = 0.98 in NHS and 0.08 in NHS II) (Table 4). In contrast, HZ was significantly associated with an elevated risk of SCD among men who were carriers of the *APOE* ε4 allele and the p-for-interaction was significant (p-interaction = 0.02). In a stratified analysis by HZ vaccination status in NHSII, there was a suggestion that the long-term risk of SCD may be greater among women who were not vaccinated against HZ (p-interaction = 0.09, NHSII) (Supplementary Table 2).

**Table 3:** Herpes zoster and RR (95% CI) of 3 unit increment in Subjective Cognitive Decline (SCD) in the Nurses’ Health Study (NHS), the Nurses’ Health Study II (NHS II), and the Health Professionals Follow-Up Study (HPFS), stratified by potentially immunocompromising conditions^*^

Years Since Herpes Zoster					
Immunocompromising Conditions (Yes/No)	Never	1–4 years	5–8 years	9–12 years	≥ 13 years
**NHS**					
Yes	(*n* = 10,473)	(*n* = 415)	(*n* = 630)	(*n* = 570)	(*n* = 413)
MV RR^a^ (95% CI)	1.00 (ref)	1.05 (0.81, 1.38)	0.84 (0.67, 1.06)	1.13 (0.90, 1.40)	1.18 (0.92, 1.53)
No	(*n* = 37,685)	(*n* = 1286)	(*n* = 1879)	(*n* = 1623)	(*n* = 1168)
MV RR^a^ (95% CI)	1.00 (ref)	1.10 (0.93, 1.29)	0.95 (0.83, 1.09)	1.01 (0.87, 1.16)	1.13 (0.96, 1.33)
	p-interaction = 0.8				
**NHS II**					
Yes	(*n* = 9392)	(*n* = 386)	(*n* = 512)	(*n* = 259)	(*n* = 791)
MV RR^a^ (95% CI)	1.00 (ref)	1.40 (1.05, 1.85)	1.03 (0.79, 1.33)	0.80 (0.56, 1.17)	1.22 (0.99, 1.50)
No	(*n* = 48,193)	(*n* = 1670)	(*n* = 2083)	(*n* = 1104)	(*n* = 2576)
MV RR^a^ (95% CI)	1.00 (ref)	1.34 (1.15, 1.55)	1.06 (0.92, 1.22)	1.12 (0.93, 1.35)	1.19 (1.06, 1.36)
	p-interaction = 0.06				
**HPFS**					
Yes	(*n* = 4469)	(*n* = 195)	(*n* = 115)	(*n* = 103)	(*n* = 149)
MV RR^b^ (95% CI)	1.00 (ref)	1.33 (0.87, 2.03)	1.13 (0.66, 1.95)	1.19 (0.66, 2.13)	1.05 (0.64, 1.73)
No	(*n* = 19,253)	(*n* = 635)	(*n* = 403)	(*n* = 334)	(*n* = 563)
MV RR^b^ (95% CI)	1.00 (ref)	1.17 (0.89, 1.54)	1.52 (1.08, 2.11)	1.50 (1.05, 2.14)	1.27 (0.96, 1.69)
	p-interaction = 0.99				

^a^Multivariable model adjusted for: age, race, family history of dementia, census tract income, husband’s education, smoking history, alcohol consumption, body mass index (BMI), physical activity, diabetes mellitus, hypertension, elevated cholesterol, Alternate Healthy Eating Index (AHEI-2010) Score, menopausal status, depression, post-menopausal hormonal therapy use, potentially immunocompromising conditions or treatments (a report of one or more of the following: cancer (other than non-melanoma skin cancer), rheumatoid arthritis (RA), Crohn’s disease/ulcerative colitis (inflammatory bowel disease), systemic lupus erythematosus (SLE), asthma, chronic obstructive pulmonary disease (COPD), oral steroids/corticosteroid use), stroke, and coronary heart disease (CHD).

^b^Multivarible model adjusted for: age, race, family history of dementia, smoking history, alcohol consumption, body mass index (BMI), physical activity, diabetes mellitus, hypertension, elevated cholesterol, Alternate Healthy Eating Index (AHEI-2010) Score, depression, profession, potentially immunocompromising conditions or treatments (a report of one or more of the following: cancer (other than non-melanoma skin cancer), rheumatoid arthritis (RA), Crohn’s disease/ulcerative colitis (inflammatory bowel disease), systemic lupus erythematosus (SLE), asthma, chronic obstructive pulmonary disease (COPD), oral steroids/corticosteroid use), stroke, and coronary heart disease (CHD)

NHS: Nurses’ Health Study

NHS II: Nurses’ Health Study II

HPFS: Health Professionals Follow-Up Study

MV RR: Multivariable-adjusted relative risk

CI: Confidence Interval

**Table 4:** Herpes zoster and RR (95% CI) of 3 unit increment in Subjective Cognitive Decline (SCD) in the Nurses’ Health Study (NHS), the Nurses’ Health Study II (NHS II), and the Health Professionals Follow-Up Study (HPFS), stratified by APOE ℇ4 carrier status

	Years Since Herpes Zoster		
APOE ℇ4 carrier status (Yes/No)	Never	1–4 years	≥ 5 years
**NHS**			
**Yes**	(*n* = 2255)	(*n* = 75)	(*n* = 328)
MV RR (95% CI)	1.00 (ref)	1.53 (0.85, 2.76)	0.96 (0.71, 1.29)
**No**	(*n* = 7110)	(*n* = 298)	(*n* = 1113)
MV RR (95% CI)	1.00 (ref)	0.80 (0.57, 1.13)	1.25 (1.06, 1.49)
	p-interaction = 0.98		
**NHSII**			
**Yes**	(*n* = 2193)	(*n* = 71)	(*n* = 364)
MV RR (95% CI)	1.00 (ref)	1.09 (0.55, 2.15)	0.87 (0.62, 1.22)
**No**	(*n* = 6650)	(*n* = 244)	(*n* = 950)
MV RR (95% CI)	1.00 (ref)	1.19 (0.80, 1.76)	1.32 (1.07, 1.62)
	p-interaction = 0.08		
**HPFS**			
**Yes**	(*n* = 1780)	(*n* = 68)	(*n* = 139)
MV RR (95% CI)	1.00 (ref)	2.83 (1.49, 5.35)	1.81 (1.10, 2.98)
**No**	(*n* = 4984)	(*n* = 213)	(*n* = 421)
MV RR (95% CI)	1.00 (ref)	1.19 (0.76, 1.86)	0.90 (0.65, 1.26)
	p-interaction = 0.02		

^a^Multivariable model adjusted for: age, race, family history of dementia, census tract income, husband’s education, smoking history, alcohol consumption, body mass index (BMI), physical activity, diabetes mellitus, hypertension, elevated cholesterol, Alternate Healthy Eating Index (AHEI-2010) Score, menopausal status, depression, post-menopausal hormonal therapy use, potentially immunocompromising conditions or treatments (a report of one or more of the following: cancer (other than non-melanoma skin cancer), rheumatoid arthritis (RA), Crohn’s disease/ulcerative colitis (inflammatory bowel disease), systemic lupus erythematosus (SLE), asthma, chronic obstructive pulmonary disease (COPD), oral steroids/corticosteroid use), stroke, and coronary heart disease (CHD).

^b^Multivarible model adjusted for: age, race, family history of dementia, smoking history, alcohol consumption, body mass index (BMI), physical activity, diabetes mellitus, hypertension, elevated cholesterol, Alternate Healthy Eating Index (AHEI-2010) Score, depression, profession, potentially immunocompromising conditions or treatments (a report of one or more of the following: cancer (other than non-melanoma skin cancer), rheumatoid arthritis (RA), Crohn’s disease/ulcerative colitis (inflammatory bowel disease), systemic lupus erythematosus (SLE), asthma, chronic obstructive pulmonary disease (COPD), oral steroids/corticosteroid use), stroke, and coronary heart disease (CHD).

NHS: Nurses’ Health Study

NHS II: Nurses’ Health Study II

HPFS: Health Professionals Follow-Up Study

MV RR: Multivariable-adjusted relative risk

CI: Confidence Interval

## Discussion

In three large independent cohorts, HZ was associated with an approximately 20% higher long-term risk of SCD. Compared to non-carriers, the risk of SCD was significantly higher among APOE ε4 carriers in men but not in women. The association between HZ and risk of SCD did not significantly differ among individuals with and without potentially immunocompromising conditions. Based on data in the NHSII, the risk of SCD following HZ may potentially be greater among those who have not been vaccinated against HZ.

To our knowledge, this is the first large study to examine HZ and the risk of early subjective cognitive decline, which may be the earliest manifestation of age-related cognitive decline and may be especially sensitive among individuals who received higher education [24]. Given the long preclinical phase of dementia [30], identifying a potential association between HZ and early cognitive decline is important as it may provide insight into the complex and possible causal relationship between viral infections and cognitive health, as well as offer opportunities for early risk reduction and improved public health strategies. Several previous studies evaluated HZ and the risk of dementia, but results have been mixed [8–10, 13, 31]. Two studies from a large health insurance database in Taiwan [7, 8] and South Korea [9] found that HZ was associated with a higher risk of Alzheimer’s disease (AD), whereas studies from the United Kingdom found no association [10, 11]. Two recent studies reported that HZ was associated with a decreased risk of dementia [12, 13], and suggested that VZV vaccination for the elderly will unlikely reduce the risk of dementia [13]. The inconsistent findings from previous studies may have arisen from the different study population and different data sources utilized, as most studies were based on retrospective reviews of insurance claims or other administrative databases using diagnostic codes, thus capturing only those individuals who sought medical attention for their HZ; misclassification of HZ using this study design has been demonstrated [32–35]. Further, the limited availability of information on health and lifestyle factors related to the risk of cognitive decline or dementia reduced the ability to adjust for these factors. The prospective cohort study design and long-term follow-up of our research enable the establishment of the temporal relationship between HZ and subsequent cognitive decline. In addition, our dataset contained detailed information on potential confounding factors, and accounting for these variables reduced the impact of confounding.

Despite some previous studies that suggesed vaccination will unlikely decrease the risk of dementia, other studies have reported the possible association between HZ vaccination and a lower risk of dementia [10, 14, 15, 36]. One retrospective study using data from the Veterans Health Administration and private sector medical claims data (MarketScan) found that HZ vaccination was associated with a 31–35% lower risk of dementia among adults aged ≥ 65 [15]. Another retrospective study in Wales also found a 28% lower risk of dementia among vaccinated adults [36]. One nested case-control study in the UK Biobank found a 20% lower dementia risk among adults who received the Zostavax vaccine [10]. Some studies also suggest that antiviral treatment may reduce the risk of dementia among individuals who had HZ [7, 37]. Findings from the current prospective cohort study, NHSII, demonstrated that the magnitude of the risk may be greater among those not vaccinated against HZ, aligning with the aforementioned literature.

There are several possible mechanisms by which HZ may contribute to cognitive decline, including neuroinflammation, cerebral vasculopathy, direct neuronal damage, and the activation of other herpesviruses like HSV-1. The inflammation induced by VZV, both locally and systemically, has been implicated in neuronal damage and accelerated cognitive decline [38]. Studies have shown increased levels of inflammatory factors in plasma exosomes of individuals with a history of HZ, suggesting a role in triggering proinflammatory responses and thrombotic processes [39].

Cerebrovascular pathologies associated with HZ, such as vasculopathy, pose another potential link to cognitive decline. This complication may lead to local inflammation, abnormal vascular remodeling, and arterial changes, increasing the risk of vascular occlusion, ischemia, and cerebrovascular disease—factors that have been shown to contribute to cognitive decline [40–42]. In the NHS, NHSII, and HPFS cohorts, we previously demonstrated that HZ was associated with up to 38% higher long-term risk of stroke, and the elevated risk persisted for 12 years or longer [43]. Plausibly, HZ is also associated with subclinical cerebrovascular changes that elevate the risk of cognitive decline.

Additionally, evidence suggests that VZV infection may contribute to amyloid-associated pathology, potentially impacting amyloid burden and disease progression in conditions like Alzheimer’s disease (AD) [44]. Moreover, HZ may indirectly accelerate cognitive decline in individuals with latent herpes simplex virus type 1 (HSV-1) [45]. HSV-1 infection has been linked to increased risk of dementia, particularly among APOE ε4 carriers [46–48], and in vitro studies suggest that VZV reactivation and neuroinflammation could induce reactivation of HSV-1, leading to AD-related changes [45].

Intriguingly, our study observed some sex differences in the relation between HZ and the risk of SCD, particularly regarding APOE ε4 carrier status. Sex differences in the relation of APOE genotype as well as other risk factors for AD, neurodegeneration, and vascular disease have been described [49–57], but whether these are due to differences in genetics, other factors, or susceptibility to AD pathology remains unclear [58, 59]. Findings regarding sex differences and the prevalence of SCD have been inconsistent [60–62], but sex-specific differences in the prevalence of certain dementia subtypes have been shown [49, 63]. Further investigation of sex differences in the relation between HZ and cognitive decline could be informative.

Strengths of the current study include the prospective cohort study design, long-term follow-up, large sample size, and comprehensive information on potential confounding factors. In addition, we have data on APOE and immunocompromising status, and HZ vaccination status was available for the NHSII, enabling us to explore potential variations in the association based on these statuses. Potential limitations of our study include self-reporting of HZ and cognitive function. However, our validation study comparing the assessment of HZ by questionnaire with medical records showed that self-reported shingles in these cohorts were highly reliable. The questions regarding subjective cognitive function used in this study have been validated against objective features of dementia and clinically established cognitive testing questionnaires [18, 26, 64–66]. Also, although we performed a validation study to determine the positive predictive value of HZ cases, which showed that 99.6% of self-reported HZ cases were true cases as confirmed by medical records, there is still the possibility of false negatives. However, if there were misclassification of HZ status, it would likely have been random and would have biased the findings towards the null or had minimal impact on the overall results due to the larger size of the non-case group. In addition, this study was observational, and residual confounding by unmeasured factors is possible. Nonetheless, our dataset has detailed information on potential confounding factors that were measured repeatedly using well-validated instruments, and the adjustment for these variables helped reduce residual confounding. Our study was limited to predominantly white healthcare professionals with generally high socioeconomic status and education, which could limit generalizability. Although this uniformity may also reduce variability and enhance internal validity of health-related information, further studies among other populations are warranted.

## Conclusion

Findings from these three large independent cohorts of women and men suggest herpes zoster was associated with a higher long-term risk of subjective cognitive decline. The risk may be greater for the APOE ε4 allele carriers among men, but not among women. The relation did not differ among those with potentially immunocompromising conditions. The magnitude of the elevated long-term risk of SCD may potentially be reduced by HZ vaccination, but further study is needed.

### Electronic supplementary material

Below is the link to the electronic supplementary material.

Supplementary Material 1

## Data Availability

Any data not published within the article will be shared at the request of other qualified investigators for purposes of replicating procedures and results. Our NHS and HPFS websites (nurseshealthstudy.org and sites.sph.harvard.edu/hpfs/) include guidelines for external users and links to all questionnaires.

## References

[1] Wolters FJ, Ikram MA. Epidemiology of dementia: the Burden on Society, the Challenges for Research. Methods Mol Biol. 2018;1750:3–14. 29512062 10.1007/978-1-4939-7704-8_1

[2] Yeh TS, Wang JD, Ku LE. Estimating Life Expectancy and Lifetime Healthcare costs for Alzheimer’s Disease in Taiwan: does the age of Disease Onset Matter? J Alzheimers Dis. 2020;73(1):307–15. 31771049 10.3233/JAD-181060

[3] Warren-Gash C, Forbes HJ, Williamson E, Breuer J, Hayward AC, Mavrodaris A, et al. Human herpesvirus infections and dementia or mild cognitive impairment: a systematic review and meta-analysis. Sci Rep. 2019;9(1):4743. 30894595 10.1038/s41598-019-41218-w PMC6426940

[4] Reynolds MA, Kruszon-Moran D, Jumaan A, Schmid DS, McQuillan GM. Varicella seroprevalence in the U.S.: data from the National Health and Nutrition Examination Survey, 1999–2004. Public Health Rep. 2010;125(6):860–9. 21121231 10.1177/003335491012500613 PMC2966667

[5] Harpaz R, Ortega-Sanchez IR, Seward JF, Advisory Committee on Immunization Practices Centers for Disease C, Prevention. Prevention of herpes zoster: recommendations of the Advisory Committee on Immunization Practices (ACIP). MMWR Recomm Rep. 2008;57(RR–5):1–30. quiz CE2-4. 18528318

[6] Anwar MM. The emerging mechanism behind viral infections and extracellular vesicles hypotheses leading to neuroinflammation and Alzheimer’s disease pathology. Ibrain. 2023;9(1):63–71. 37786515 10.1002/ibra.12090 PMC10529198

[7] Chen VC, Wu SI, Huang KY, Yang YH, Kuo TY, Liang HY et al. Herpes zoster and dementia: a Nationwide Population-based Cohort Study. J Clin Psychiatry. 2018;79(1). 10.4088/JCP.16m11312 29244265

[8] Tsai MC, Cheng WL, Sheu JJ, Huang CC, Shia BC, Kao LT, et al. Increased risk of dementia following herpes zoster ophthalmicus. PLoS ONE. 2017;12(11):e0188490. 29166672 10.1371/journal.pone.0188490 PMC5699837

[9] Shim Y, Park M, Kim J. Increased incidence of dementia following herpesvirus infection in the Korean population. Med (Baltim). 2022;101(41):e31116. 10.1097/MD.0000000000031116 PMC9575754 36254002

[10] Lophatananon A, Mekli K, Cant R, Burns A, Dobson C, Itzhaki R, et al. Shingles, Zostavax vaccination and risk of developing dementia: a nested case-control study-results from the UK Biobank cohort. BMJ Open. 2021;11(10):e045871. 34625411 10.1136/bmjopen-2020-045871 PMC8504358

[11] Warren-Gash C, Williamson E, Shiekh SI, Borjas-Howard J, Pearce N, Breuer JM, et al. No evidence that herpes zoster is associated with increased risk of dementia diagnosis. Ann Clin Transl Neurol. 2022;9(3):363–74. 35170873 10.1002/acn3.51525 PMC8935278

[12] Choi HG, Park BJ, Lim JS, Sim SY, Jung YJ, Lee SW. Herpes Zoster does not increase the risk of neurodegenerative dementia: a case-control study. Am J Alzheimers Dis Other Demen. 2021;36:15333175211006504. 33882722 10.1177/15333175211006504 PMC11005322

[13] Schmidt SAJ, Veres K, Sørensen HT, Obel N, Henderson VW. Incident herpes zoster and risk of dementia: a Population-based Danish cohort study. Neurology. 2022;99(7):e660–8. 35676090 10.1212/WNL.0000000000200709 PMC9484607

[14] Harris K, Ling Y, Bukhbinder AS, Chen L, Phelps KN, Cruz G, et al. The impact of routine vaccinations on Alzheimer’s disease risk in persons 65 years and older: a claims-based cohort study using propensity score matching. J Alzheimers Dis. 2023;95(2):703–18. 10.3233/JAD-221231 PMC10578243 37574727

[15] Scherrer JF, Salas J, Wiemken TL, Hoft DF, Jacobs C, Morley JE. Impact of herpes zoster vaccination on incident dementia: a retrospective study in two patient cohorts. PLoS ONE. 2021;16(11):e0257405. 34788293 10.1371/journal.pone.0257405 PMC8597989

[16] Lovheim H, Norman T, Weidung B, Olsson J, Josefsson M, Adolfsson R, et al. Herpes Simplex Virus, APOEvarepsilon4, and Cognitive decline in Old Age: results from the Betula Cohort Study. J Alzheimers Dis. 2019;67(1):211–20. 30636735 10.3233/JAD-171162

[17] Linard M, Letenneur L, Garrigue I, Doize A, Dartigues JF, Helmer C. Interaction between APOE4 and herpes simplex virus type 1 in Alzheimer’s disease. Alzheimers Dement. 2020;16(1):200–8. 31914220 10.1002/alz.12008

[18] Donovan NJ, Amariglio RE, Zoller AS, Rudel RK, Gomez-Isla T, Blacker D, et al. Subjective cognitive concerns and neuropsychiatric predictors of progression to the early clinical stages of Alzheimer disease. Am J Geriatr Psychiatry. 2014;22(12):1642–51. 24698445 10.1016/j.jagp.2014.02.007 PMC4145054

[19] Duara R, Loewenstein DA, Greig MT, Potter E, Barker W, Raj A, et al. Pre-MCI and MCI: neuropsychological, clinical, and imaging features and progression rates. Am J Geriatr Psychiatry. 2011;19(11):951–60. 21422909 10.1097/JGP.0b013e3182107c69 PMC3175279

[20] Ulbl J, Rakusa M. The Importance of Subjective Cognitive Decline Recognition and the Potential of Molecular and Neurophysiological Biomarkers&mdash;A Systematic Review. International Journal of Molecular Sciences. 2023;24(12):10158. 10.3390/ijms241210158 PMC10299427 37373304

[21] Parfenov VA, Zakharov VV, Kabaeva AR, Vakhnina NV. Subjective cognitive decline as a predictor of future cognitive decline: a systematic review. Dement Neuropsychol. 2020;14(3):248–57. 32973979 10.1590/1980-57642020dn14-030007 PMC7500809

[22] Samieri C, Proust-Lima C, M MG, Okereke OI, Amariglio RE, Sperling RA, et al. Subjective cognitive concerns, episodic memory, and the APOE epsilon4 allele. Alzheimer’s Dement J Alzheimer’s Assoc. 2014;10(6):752–e91. 10.1016/j.jalz.2014.06.012 PMC4253880 25256133

[23] Yuan C, Fondell E, Bhushan A, Ascherio A, Okereke OI, Grodstein F, et al. Long-term intake of vegetables and fruits and subjective cognitive function in US men. Neurology. 2019;92(1):e63–75. 30464030 10.1212/WNL.0000000000006684 PMC6336164

[24] van Oijen M, de Jong FJ, Hofman A, Koudstaal PJ, Breteler MM. Subjective memory complaints, education, and risk of Alzheimer’s disease. Alzheimers Dement. 2007;3(2):92–7. 19595922 10.1016/j.jalz.2007.01.011

[25] Yeh TS, Yuan C, Ascherio A, Rosner BA, Willett WC, Blacker D. Long-term Dietary Flavonoid Intake and Subjective Cognitive decline in US men and women. Neurology. 2021;97(10):e1041–56. 34321362 10.1212/WNL.0000000000012454 PMC8448553

[26] Amariglio RE, Townsend MK, Grodstein F, Sperling RA, Rentz DM. Specific subjective memory complaints in older persons may indicate poor cognitive function. J Am Geriatr Soc. 2011;59(9):1612–7. 21919893 10.1111/j.1532-5415.2011.03543.x PMC3315361

[27] Yeh TS, Yuan C, Ascherio A, Rosner BA, Blacker D, Willett WC. Long-term dietary protein intake and subjective cognitive decline in US men and women. Am J Clin Nutr. 2022;115(1):199–210. 34293099 10.1093/ajcn/nqab236 PMC8755047

[28] Yeh TS, Yuan C, Ascherio A, Rosner BA, Blacker D, Willett WC. Long-term intake of total energy and fat in relation to subjective cognitive decline. Eur J Epidemiol. 2022;37(2):133–46. 34748116 10.1007/s10654-021-00814-9 PMC8960333

[29] Kang JH, Logroscino G, De Vivo I, Hunter D, Grodstein F. Apolipoprotein E, cardiovascular disease and cognitive function in aging women. Neurobiol Aging. 2005;26(4):475–84. 15653176 10.1016/j.neurobiolaging.2004.05.003

[30] Dubois B, Hampel H, Feldman HH, Scheltens P, Aisen P, Andrieu S, et al. Preclinical Alzheimer’s disease: definition, natural history, and diagnostic criteria. Alzheimers Dement. 2016;12(3):292–323. 27012484 10.1016/j.jalz.2016.02.002 PMC6417794

[31] Levine KS, Leonard HL, Blauwendraat C, Iwaki H, Johnson N, Bandres-Ciga S et al. Virus exposure and neurodegenerative disease risk across national biobanks. Neuron. 2023. 10.1016/j.neuron.2022.12.029 PMC10079561 36669485

[32] Kiyota Y, Schneeweiss S, Glynn RJ, Cannuscio CC, Avorn J, Solomon DH. Accuracy of Medicare claims-based diagnosis of acute myocardial infarction: estimating positive predictive value on the basis of review of hospital records. Am Heart J. 2004;148(1):99–104. 15215798 10.1016/j.ahj.2004.02.013

[33] Cheng CL, Kao YH, Lin SJ, Lee CH, Lai ML. Validation of the National Health Insurance Research Database with ischemic stroke cases in Taiwan. Pharmacoepidemiol Drug Saf. 2011;20(3):236–42. 21351304 10.1002/pds.2087

[34] Yawn BP, Wollan P, St Sauver J. Comparing shingles incidence and complication rates from medical record review and administrative database estimates: how close are they? Am J Epidemiol. 2011;174(9):1054–61. 21920944 10.1093/aje/kwr206 PMC3243933

[35] Erskine N, Tran H, Levin L, Ulbricht C, Fingeroth J, Kiefe C, et al. A systematic review and meta-analysis on herpes zoster and the risk of cardiac and cerebrovascular events. PLoS ONE. 2017;12(7):e0181565. 28749981 10.1371/journal.pone.0181565 PMC5531458

[36] Schnier C, Janbek J, Lathe R, Haas J. Reduced dementia incidence after varicella zoster vaccination in Wales 2013–2020. Alzheimers Dement (N Y). 2022;8(1):e12293. 35434253 10.1002/trc2.12293 PMC9006884

[37] Bae S, Yun SC, Kim MC, Yoon W, Lim JS, Lee SO, et al. Association of herpes zoster with dementia and effect of antiviral therapy on dementia: a population-based cohort study. Eur Arch Psychiatry Clin Neurosci. 2021;271(5):987–97. 32613564 10.1007/s00406-020-01157-4

[38] Wang JP, Kurt-Jones EA, Shin OS, Manchak MD, Levin MJ, Finberg RW. Varicella-Zoster virus activates inflammatory cytokines in human monocytes and macrophages via toll-like receptor 2. J Virol. 2005;79(20):12658–66. 16188968 10.1128/JVI.79.20.12658-12666.2005 PMC1235827

[39] Bubak AN, Coughlan C, Posey J, Saviola AJ, Niemeyer CS, Lewis SWR et al. Zoster-associated prothrombotic plasma exosomes and increased stroke risk. J Infect Dis. 2022. 10.1093/infdis/jiac405 PMC10319974 36200236

[40] O’Brien JT, Erkinjuntti T, Reisberg B, Roman G, Sawada T, Pantoni L, et al. Vascular cognitive impairment. Lancet Neurol. 2003;2(2):89–98. 12849265 10.1016/S1474-4422(03)00305-3

[41] Pendlebury ST, Rothwell PM, Oxford Vascular S. Incidence and prevalence of dementia associated with transient ischaemic attack and stroke: analysis of the population-based Oxford Vascular Study. Lancet Neurol. 2019;18(3):248–58. 30784556 10.1016/S1474-4422(18)30442-3 PMC6390174

[42] Tatemichi TK, Desmond DW, Mayeux R, Paik M, Stern Y, Sano M, et al. Dementia after stroke: baseline frequency, risks, and clinical features in a hospitalized cohort. Neurology. 1992;42(6):1185–93. 1603346 10.1212/WNL.42.6.1185

[43] Curhan SG, Kawai K, Yawn B, Rexrode KM, Rimm EB, Curhan GC. Herpes zoster and long-term risk of Cardiovascular Disease. J Am Heart Assoc. 2022;11(23):e027451. 36382961 10.1161/JAHA.122.027451 PMC9851464

[44] Mescher T, Boyer PJ, Bubak AN, Hassell JE Jr., Nagel MA. Detection of varicella zoster virus antigen and DNA in two cases of cerebral amyloid angiopathy. J Neurol Sci. 2021;422:117315. 33503519 10.1016/j.jns.2021.117315 PMC7935758

[45] Cairns DM, Itzhaki RF, Kaplan DL. Potential involvement of Varicella Zoster Virus in Alzheimer’s Disease via Reactivation of quiescent herpes simplex virus type 1. J Alzheimers Dis. 2022;88(3):1189–200. 35754275 10.3233/JAD-220287

[46] Ou YN, Zhu JX, Hou XH, Shen XN, Xu W, Dong Q, et al. Associations of Infectious agents with Alzheimer’s Disease: a systematic review and Meta-analysis. J Alzheimers Dis. 2020;75(1):299–309. 32280095 10.3233/JAD-191337

[47] Sochocka M, Zwolinska K, Leszek J. The infectious etiology of Alzheimer’s Disease. Curr Neuropharmacol. 2017;15(7):996–1009. 28294067 10.2174/1570159X15666170313122937 PMC5652018

[48] Damiano RF, Guedes BF, de Rocca CC, de Padua Serafim A, Castro LHM, Munhoz CD, et al. Cognitive decline following acute viral infections: literature review and projections for post-COVID-19. Eur Arch Psychiatry Clin Neurosci. 2022;272(1):139–54. 34173049 10.1007/s00406-021-01286-4 PMC8231753

[49] Kim MY, Kim K, Hong CH, Lee SY, Jung YS. Sex differences in Cardiovascular Risk factors for Dementia. Biomol Ther (Seoul). 2018;26(6):521–32. 30464071 10.4062/biomolther.2018.159 PMC6254640

[50] Lahoz C, Schaefer EJ, Cupples LA, Wilson PW, Levy D, Osgood D, et al. Apolipoprotein E genotype and cardiovascular disease in the Framingham Heart Study. Atherosclerosis. 2001;154(3):529–37. 11257253 10.1016/S0021-9150(00)00570-0

[51] Altmann A, Tian L, Henderson VW, Greicius MD. Alzheimer’s Disease Neuroimaging Initiative I. Sex modifies the APOE-related risk of developing Alzheimer disease. Ann Neurol. 2014;75(4):563–73. 24623176 10.1002/ana.24135 PMC4117990

[52] Ungar L, Altmann A, Greicius MD. Apolipoprotein E, gender, and Alzheimer’s disease: an overlooked, but potent and promising interaction. Brain Imaging Behav. 2014;8(2):262–73. 24293121 10.1007/s11682-013-9272-x PMC4282773

[53] Hohman TJ, Dumitrescu L, Barnes LL, Thambisetty M, Beecham G, Kunkle B, et al. Sex-Specific Association of Apolipoprotein E with cerebrospinal fluid levels of tau. JAMA Neurol. 2018;75(8):989–98. 29801024 10.1001/jamaneurol.2018.0821 PMC6142927

[54] Yaffe K, Haan M, Byers A, Tangen C, Kuller L. Estrogen use, APOE, and cognitive decline: evidence of gene-environment interaction. Neurology. 2000;54(10):1949–54. 10822435 10.1212/WNL.54.10.1949

[55] Kang JH, Grodstein F. Postmenopausal hormone therapy, timing of initiation, APOE and cognitive decline. Neurobiol Aging. 2012;33(7):1129–37. 21122949 10.1016/j.neurobiolaging.2010.10.007 PMC3483632

[56] Hogervorst E, Lehmann DJ, Warden DR, McBroom J, Smith AD. Apolipoprotein E epsilon4 and testosterone interact in the risk of Alzheimer’s disease in men. Int J Geriatr Psychiatry. 2002;17(10):938–40. 12325053 10.1002/gps.714

[57] Pfankuch T, Rizk A, Olsen R, Poage C, Raber J. Role of circulating androgen levels in effects of apoE4 on cognitive function. Brain Res. 2005;1053(1–2):88–96. 16054121 10.1016/j.brainres.2005.06.028

[58] Carter CL, Resnick EM, Mallampalli M, Kalbarczyk A. Sex and gender differences in Alzheimer’s disease: recommendations for future research. J Womens Health (Larchmt). 2012;21(10):1018–23. 22917473 10.1089/jwh.2012.3789

[59] Hebert LE, Weuve J, Scherr PA, Evans DA. Alzheimer disease in the United States (2010–2050) estimated using the 2010 census. Neurology. 2013;80(19):1778–83. 23390181 10.1212/WNL.0b013e31828726f5 PMC3719424

[60] Brown MJ, Patterson R. Subjective cognitive decline among sexual and gender minorities: results from a U.S. Population-based sample. J Alzheimers Dis. 2020;73(2):477–87. 31796675 10.3233/JAD-190869 PMC7299090

[61] Lee SH, Kang Y, Cho SJ. Subjective cognitive decline in patients with migraine and its relationship with depression, anxiety, and sleep quality. J Headache Pain. 2017;18(1):77. 28744704 10.1186/s10194-017-0779-1 PMC5526827

[62] Schliep KC, Barbeau WA, Lynch KE, Sorweid MK, Varner MW, Foster NL, et al. Overall and sex-specific risk factors for subjective cognitive decline: findings from the 2015–2018 behavioral risk factor Surveillance System Survey. Biology Sex Differences. 2022;13(1):16. 10.1186/s13293-022-00425-3 PMC9004039 35414037

[63] Akhter F, Persaud A, Zaokari Y, Zhao Z, Zhu D. Vascular dementia and underlying sex differences. Front Aging Neurosci. 2021;13:720715. 34566624 10.3389/fnagi.2021.720715 PMC8457333

[64] Samieri C, Proust-Lima C, M MG, Okereke OI, Amariglio RE, Sperling RA, et al. Subjective cognitive concerns, episodic memory, and the APOE epsilon4 allele. Alzheimers Dement. 2014;10(6):752–e91. 25256133 10.1016/j.jalz.2014.06.012 PMC4253880

[65] Amariglio RE, Becker JA, Carmasin J, Wadsworth LP, Lorius N, Sullivan C, et al. Subjective cognitive complaints and amyloid burden in cognitively normal older individuals. Neuropsychologia. 2012;50(12):2880–6. 22940426 10.1016/j.neuropsychologia.2012.08.011 PMC3473106

[66] Amariglio RE, Mormino EC, Pietras AC, Marshall GA, Vannini P, Johnson KA, et al. Subjective cognitive concerns, amyloid-beta, and neurodegeneration in clinically normal elderly. Neurology. 2015;85(1):56–62. 26048028 10.1212/WNL.0000000000001712 PMC4501939

