# Supplementary material for: Herpes zoster and long-term risk of subjective cognitive decline
Source: Alzheimers Res Ther. 2024 Aug 14;16:180. doi: 10.1186/s13195-024-01511-x (PMC11323373; doi:10.1186/s13195-024-01511-x)
Supplement: Supplementary file 1 — Supplementary Material 1 [file 13195_2024_1511_MOESM1_ESM.doc]

**Herpes Zoster and Long-Term Risk of Subjective Cognitive Decline**

Tian-Shin Yeh, MD, PhD^1,2,3,4,5*^; Gary C. Curhan, MD, ScD^4,6,7^; Barbara P. Yawn, MD, MSc^8^; Walter C. Willett, MD, DrPH ^3^; Sharon G. Curhan, MD, ScM ^4,6^

^1^Department of Physical Medicine and Rehabilitation, School of Medicine, College of Medicine, Taipei Medical University, Taipei, Taiwan

^2^Department of Physical Medicine and Rehabilitation, Wan Fang Hospital, Taipei Medical University, Taipei, Taiwan

^3^Department of Epidemiology and Nutrition, Harvard T. H. Chan School of Public Health, Harvard University, Boston, MA, USA

^4^Channing Division of Network Medicine, Department of Medicine, Brigham and Women’s Hospital, Boston, MA, USA

^5^Department of Physical Medicine and Rehabilitation, National Taiwan University Hospital, Taipei, Taiwan; Department of Physical Medicine and Rehabilitation, College of Medicine, National Taiwan University, Taipei, Taiwan

^6^Harvard Medical School, Boston, MA, USA

^7^Renal Division, Department of Medicine, Brigham and Women’s Hospital, Boston, MA, USA

^8^Department of Family and Community Health, University of Minnesota, Minneapolis, MN, USA

**Short running head**: Herpes Zoster and Subjective Cognitive Decline

**Corresponding author:**

Tian-Shin Yeh, MD, PhD

Department of Physical Medicine and Rehabilitation, School of Medicine, College of Medicine, Taipei Medical University, No.250, Wuxing St., Taipei 11031, Taiwan; Phone：02-2736-1661

email: tianshin.yeh@gmail.com

NHS: HPFS:


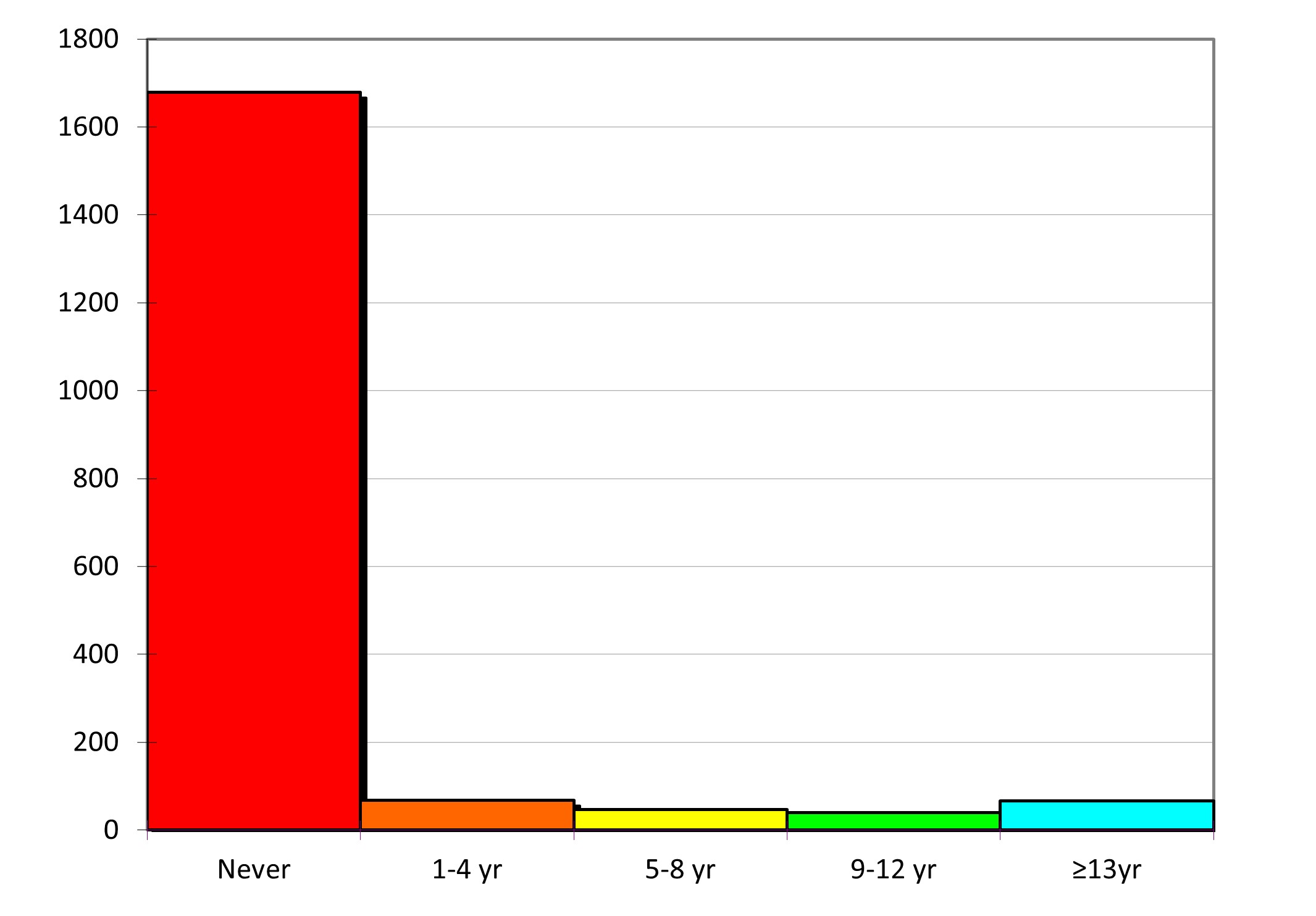

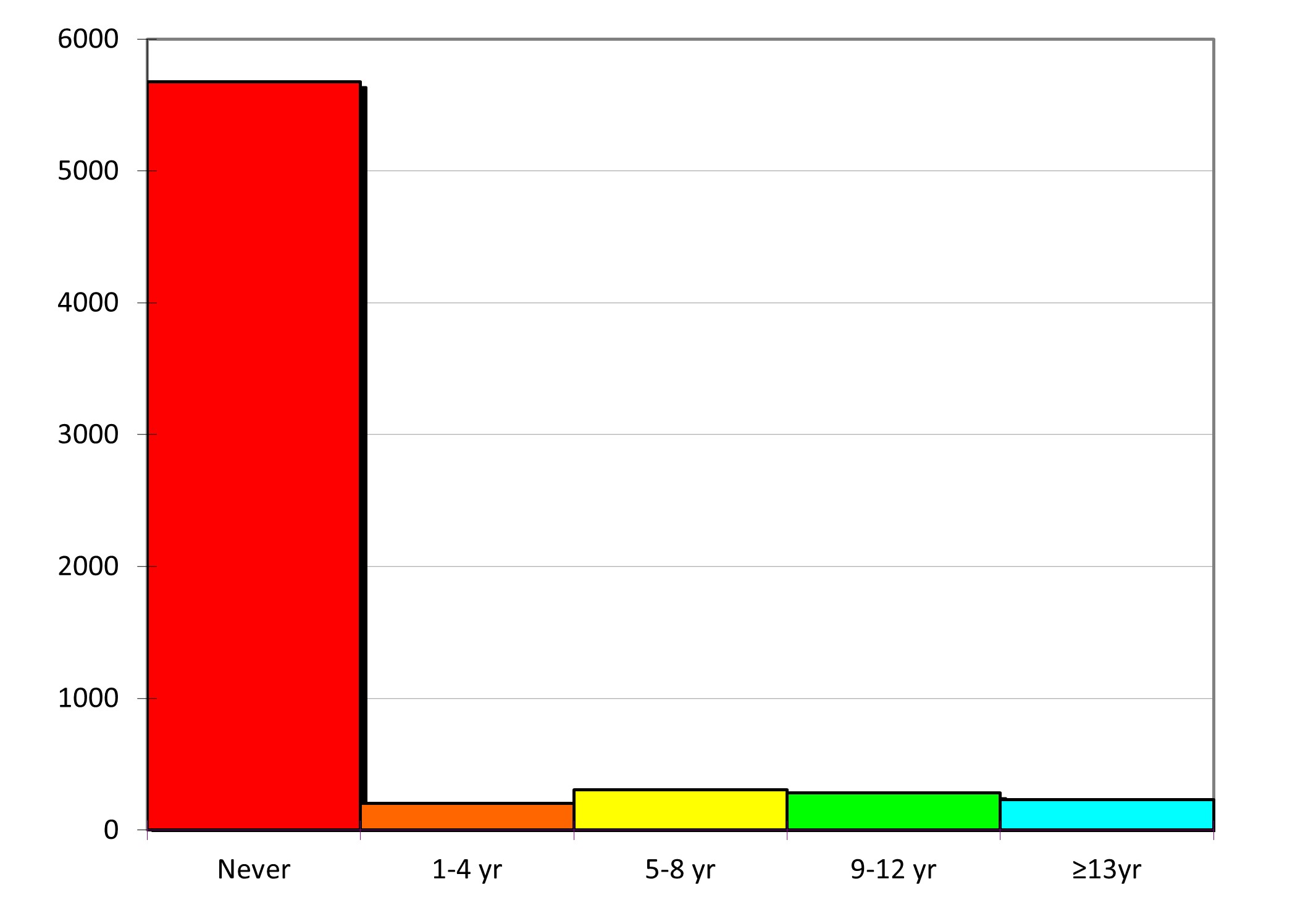


NHSII:


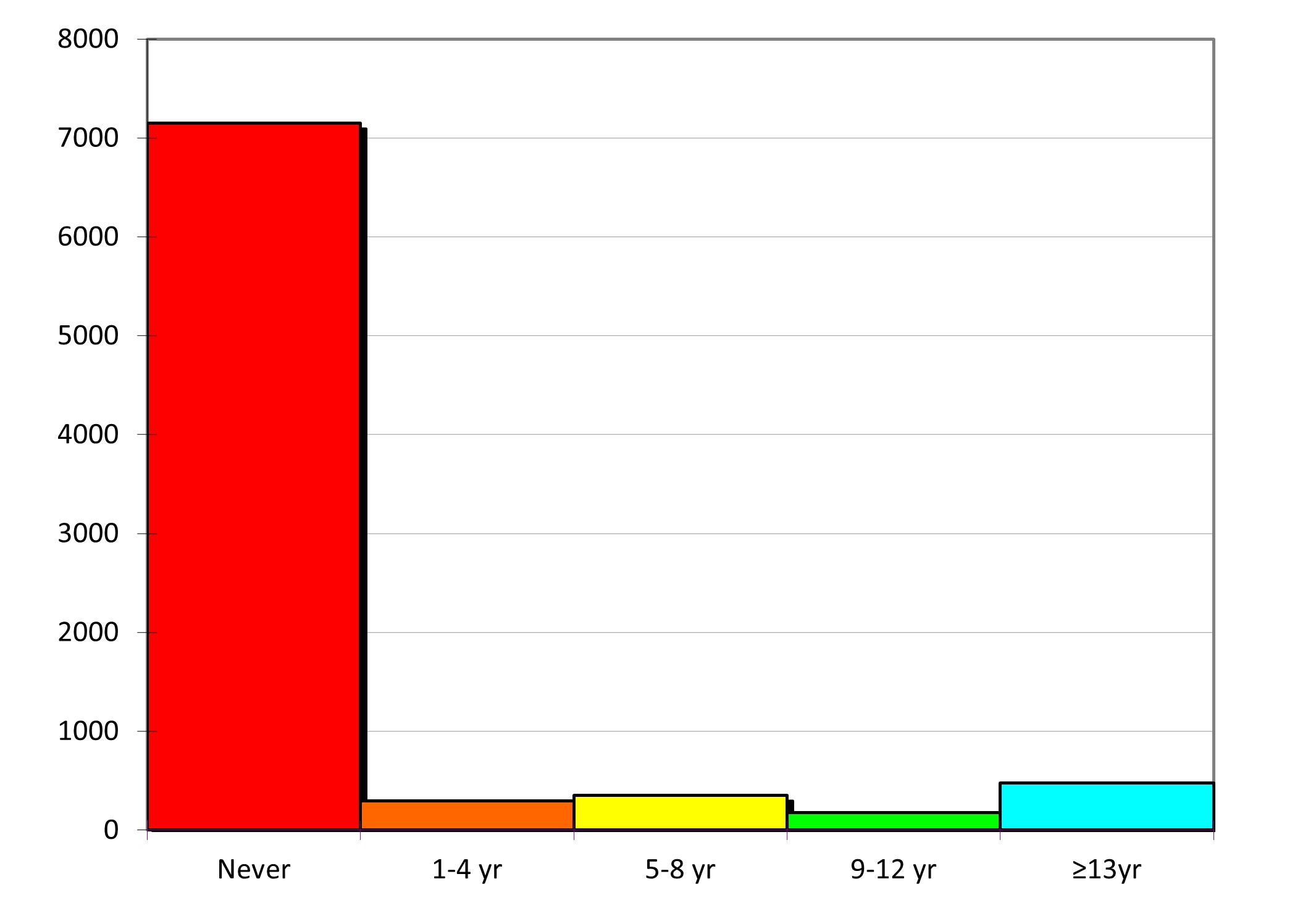


**Supplementary Figure 1:** The frequencies of participants with SCD>=3 by time since HZ in the Nurses’ Health Study (NHS), the Nurses’ Health Study II (NHS II), and the Health Professionals Follow-up Study (HPFS)

**Supplementary Table 1: Effect of adjustment for covariates on the association between Herpes Zoster and 3 unit increment in Subjective Cognitive Decline (SCD) in the Nurses’ Health Study (NHS), the Nurses’ Health Study II (NHS II), and the Health Professionals Follow-up Study (HPFS)**

|  | **Years Since Herpes Zoster** | | | | |
| --- | --- | --- | --- | --- | --- |
| *Incremental adjustment* | **Never** | **1-4 years** | **5-8 years** | **9-12 years** | **≥13 years** |
| **NHS** |  |  |  |  |  |
| Age | 1.00 (ref) | 1.15 (0.99, 1.32) | 0.96 (0.86, 1.08) | 1.09 (0.97, 1.23) | 1.22 (1.06, 1.40) |
| + CHD | 1.00 (ref) | 1.15 (1.00, 1.32) | 0.96 (0.86, 1.08) | 1.09 (0.97, 1.23) | 1.22 (1.06, 1.40) |
| + Stroke | 1.00 (ref) | 1.14 (0.99, 1.31) | 0.96 (0.86, 1.08) | 1.09 (0.97, 1.23) | 1.22 (1.07, 1.40) |
| + depression | 1.00 (ref) | 1.11 (0.97, 1.28) | 0.95 (0.85, 1.07) | 1.05 (0.93, 1.18) | 1.19 (1.04, 1.36) |
| **NHSII** |  |  |  |  |  |
| Age | 1.00 (ref) | 1.42 (1.25, 1.63) | 1.10 (0.97, 1.25) | 1.12 (0.95, 1.33) | 1.38 (1.24, 1.53) |
| + CHD | 1.00 (ref) | 1.43 (1.25, 1.63) | 1.10 (0.97, 1.24) | 1.12 (0.95, 1.32) | 1.37 (1.23, 1.52) |
| + Stroke | 1.00 (ref) | 1.43 (1.25, 1.63) | 1.10 (0.97, 1.24) | 1.12 (0.95, 1.32) | 1.37 (1.23, 1.52) |
| + depression | 1.00 (ref) | 1.38 (1.21, 1.58) | 1.08 (0.96, 1.23) | 1.05 (0.89, 1.24) | 1.28 (1.15, 1.42) |
| **HPFS** |  |  |  |  |  |
| Age | 1.00 (ref) | 1.29 (1.03, 1.62) | 1.47 (1.11, 1.95) | 1.61 (1.19, 2.18) | 1.42 (1.11, 1.82) |
| + CHD | 1.00 (ref) | 1.27 (1.00, 1.59) | 1.47 (1.11, 1.96) | 1.57 (1.16, 2.13) | 1.41 (1.11, 1.81) |
| + Stroke | 1.00 (ref) | 1.27 (1.00, 1.60) | 1.49 (1.12, 1.98) | 1.58 (1.17, 2.15) | 1.42 (1.12, 1.82) |
| + depression | 1.00 (ref) | 1.26 (1.00, 1.59) | 1.42 (1.07, 1.88) | 1.53 (1.13, 2.08) | 1.31 (1.03, 1.67) |

**Supplementary Table 2: Herpes Zoster and RR (95% CI) of 3 unit increment in Subjective Cognitive Decline (SCD) in the Nurses’ Health Study II (NHS II)), stratified by herpes zoster vaccination status**

| **Years Since Herpes Zoster** | | | | | |
| --- | --- | --- | --- | --- | --- |
| **HZ vaccination status (Yes/No)** | **Never** | **1-4 years** | **5-8 years** | **9-12 years** | **≥13 years** |
| **NHS II** |  |  |  |  |  |
| **Yes** | (n=17750) | (n=718) | (n=966) | (n=495) | (n=1076) |
| MV RR^a^ (95% CI) | 1.00 (ref) | 1.24 (0.99, 1.55) | 1.11 (0.91, 1.36) | 1.12 (0.85, 1.47) | 1.09 (0.91, 1.32) |
| **No** | (n=39835) | (n=1338) | (n=1629) | (n=868) | (n=2291) |
| MV RR^a^ (95% CI) | 1.00 (ref) | 1.40 (1.19, 1.66) | 1.01 (0.86, 1.19) | 1.01 (0.82, 1.25) | 1.25 (1.10, 1.43) |
|  | p-interaction = 0.09 | | | | |

^a^Multivariable model adjusted for: age, race, family history of dementia, census tract income, husband’s education, smoking history, alcohol consumption, body mass index (BMI), physical activity, diabetes mellitus, hypertension, elevated cholesterol, AHEI-2010 Score, menopausal status, depression, post-menopausal hormonal therapy use, potentially immunocompromising conditions or treatments (a report of one or more of the following: cancer (other than non-melanoma skin cancer), rheumatoid arthritis (RA), Crohn’s disease/ulcerative colitis (inflammatory bowel disease), systemic lupus erythematosus (SLE), asthma, chronic obstructive pulmonary disease (COPD), oral steroids/corticosteroid use), stroke, and CHD.

NHS II: Nurses’ Health Study II

MV RR: Multivariable-adjusted relative risk

CI: Confidence Interval
